# Metabolic Profiles and Free Radical Scavenging Activity of *Cordyceps bassiana* Fruiting Bodies According to Developmental Stage

**DOI:** 10.1371/journal.pone.0073065

**Published:** 2013-09-13

**Authors:** Sun-Hee Hyun, Seok-Young Lee, Gi-Ho Sung, Seong Hwan Kim, Hyung-Kyoon Choi

**Affiliations:** 1 College of Pharmacy, Chung-Ang University, Seoul, Republic of Korea; 2 Mushroom Research Division, Department of Herbal Crop Research, National Institute of Horticultural and Herbal Science, RDA, Suwon, Republic of Korea; 3 Department of Microbiology, Dankook University, Cheonan, Republic of Korea; University of Wisconsin – Madison, United States of America

## Abstract

The metabolic profiles of *Cordyceps bassiana* according to fruiting body developmental stage were investigated using gas chromatography-mass spectrometry. We were able to detect 62 metabolites, including 48 metabolites from 70% methanol extracts and 14 metabolites from 100% *n*-hexane extracts. These metabolites were classified as alcohols, amino acids, organic acids, phosphoric acids, purine nucleosides and bases, sugars, saturated fatty acids, unsaturated fatty acids, or fatty amides. Significant changes in metabolite levels were found according to developmental stage. Relative levels of amino acids, purine nucleosides, and sugars were higher in development stage 3 than in the other stages. Among the amino acids, valine, isoleucine, lysine, histidine, glutamine, and aspartic acid, which are associated with ABC transporters and aminoacyl-tRNA biosynthesis, also showed higher levels in stage 3 samples. The free radical scavenging activities, which were significantly higher in stage 3 than in the other stages, showed a positive correlation with purine nucleoside metabolites such as adenosine, guanosine, and inosine. These results not only show metabolic profiles, but also suggest the metabolic pathways associated with fruiting body development stages in cultivated *C. bassiana*.

## Introduction

Fungi are used extensively in the production of a broad range of metabolites, such as pharmaceuticals, enzymes, and toxins for plants, animals, and humans [1], [2]. *Cordyceps bassiana*, belonging to the Ascomycetes, is known to be an entomopathogenic fungus for insects and other arthropod pests with a broad host range, and *C. bassiana* was found growing in a Lepidopteran cadaver in the wild [3]–[6]. In addition, *C. bassiana* was recently successfully cultured *in vitro* for commercial use [7], [8]. Various compounds including polysaccharides, ergosterol, mannitol, beauvericin, bassiacridin, bassianolide, beauverolide, and enniatin have been identified in *C. bassiana* [9]–[11].This fungi is known to have various bioactivities such as antiinflammatory [12], [13], antioxidant [14],antibiotic [15], and atopic dermatitis regulatory activities [16].

A metabolomic approach has been used for major metabolite identification in fungus as well as for fungal taxonomy of *Penicillium* using mass spectrometry [17], [18] and for characterization of secondary metabolites of *Aspergillus* [19].Only one metabolic profiling study on *C. bassiana* during development using nuclear magnetic resonance spectrometry has recently been reported [20].However, the investigation of the major metabolites and metabolism associated with and the free-radical scavenging activities of cultivated *C. bassiana* have not yet been elucidated. Moreover, most studies on *Cordyceps* species during fruiting body formation have only focused on targeted chemical composition change [21] and gene expression profiling [22], [23].

The enrichment analysis method was recently developed for the functional interpretation of large amounts of data in the fields of genomics, transcriptomics, proteomics, and metabolomics [24], [25]. Enrichment analysis is a useful tool to investigate wide ranges of biological and chemical annotations in several organisms [26]. Recently, studies on biomarker annotation in human liver tissue, metabolomic correlation networks in *Arabidopsis*, and a global test for metabolic pathways in *Escherichia coli* and *Saccharomyces cerevisiae* under different conditions have been reported by functional enrichment analysis [27]–[29].

However, to the best of our knowledge, no research using enrichment analysis has investigated metabolite annotation or metabolism changes in *C. bassiana* according to fruiting bodies, which are classified from stages 1 to 4 during the formation of stromata and perithecium of fruiting body surfaces (stage 1,prior to perithecium formations; stage 2,early perithecium formation; stage 3,completed perithecium formation; and stage 4,aging after perithecium formation). Perithecia that from the stromata are flask-shaped structures containing ascospores, and the characteristic morphology of *C. bassiana* stromata has been reported previously [8].Thus, the developmental stages of *C. bassiana* fruiting bodies were categorized by the degree of perithecium formation in this study.

The main hypothesis is that the metabolite level associated with specific metabolisms and free-radical scavenging activity might change according to developmental stage of cultivated *C. bassiana*. In this study, we performed metabolic profiling of cultivated *C. bassiana* in various developmental stages using gas chromatography–mass spectrometry (GC-MS). In addition, the free-radical scavenging activities of those samples and their correlation with specific metabolites were investigated. The main objectives of this study were metabolic profiling and investigation of the free-radical scavenging activities in cultivated *C. bassiana* at various developmental stages. The major metabolic pathways associated with developmental stages will also be discussed.

## Materials and Methods

### Sample preparation of fruiting body

The anamorph of *C. bassiana* is *B. bassiana*, and the nuclear intergenic region Bloc and the nuclear ribosomal internal transcribed spacer region (ITS) were used to confirm the isolates used for fruiting body development in this study were *C. bassiana* based on the recent phylogenetic analyses of Rehner et al [30].For the artificial production of fruiting bodies, *C. bassiana* strains were grown on Sabouraud dextrose +1% (w/v) yeast extract broth (SDY) for 3 days at 25°C as inocula for the production of fruiting bodies of *C. bassiana*. The cultures of fruiting body were grown in 1,000-ml plastic bottles containing brown rice medium and incubated at 20°C under light intensity of 400 lux and 90% humidity. Samples were collected every week from the fourth to seventh weeks of cultivation to monitor and compare the development of stromata and perithecia. Observations were made using a dissecting microscope (SZ2-1ILST, Olympus, Japan) and the four developmental stages were classified as follows: stage 1, week 4 (no perithecia formation on any stroma); stage 2, week 5 (initiation of perithecium formation, yellowish tiny spots appear on the stromata); stage 3, week 6 (club-shaped perithecium formation, filiform ascospores produced in the perithecium); stage 4, week 7 (aging stage, ascospores released from the perithecia). The fruiting bodies isolated from culture media were freeze-dried and powdered according to developmental stage. Samples were stored at −70°C before analysis. Voucher specimens were deposited at the College of Pharmacy, Chung-Ang University, Republic of Korea (fruiting bodies: CAUCBF 20110904–20110907).

### Sample preparation for GC-MS analysis

Methanol and n-hexane were used as solvents for the extraction of polar and nonpolar metabolites, respectively. Twenty milligrams of each sample in different growth conditions were transferred into glass Eppendorf tubes (Axygen, Union City, CA) and extracted with 1 mL of 70% methanol and 100% *n*-hexane. After sonication, the tube was centrifuged at 2,000 rpm for 10 min. The supernatant was collected separately from each sample and filtered through a 0.45-um filter (Acrodisc Syringe Filters, Pall Corporation, NY). After extraction, 100 µL of each sample solution was separately transferred into GC vials and then dried with nitrogen gas flow for 5 min at 60°C. After drying, 30 µL of 20,000 µg/mL methoxylamine hydrochloride in pyridine was added. Thereafter, 50 µL of BSTFA (N,O-bis (trimethylsilyl) trifluoroacetamide; Alfa Aesar, Ward Hill, Massachusetts) containing 1% TMCS (trimethyl chlorosilane) and 10 µL of 2-chloronaphthalene (Tokyo Chemical Industry Co., Ltd., Tokyo, Japan; 250 µg/mL in pyridine as an internal standard) were added to the dried sample. Derivatized samples were incubated at 60°C for 60 min, after which the solutions were directly used for GC-MS analysis.For quantification of purines (adenosine, guanosine, inosine, uric acid) in *C. bassiana* samples, standard solutions (1–100 µg/mL) and sample solution (10,000 mg/L) were prepared with 70% methanol. The sample and each standard solution of 90 µL were transferred into GC vial that was dried with nitrogen gas for 5 min at 60°C.The derivatization was performed as above described method. After derivatization process, the solution was directly used for GC-MS analysis. Characteristic ions of adenosine (230 m/z), guanosine (324 m/z), inosine (217 m/z), and uric acid (411 m/z) were selected in preliminary analysis, and those were used for each purine quantification of *C. bassiana* fruiting bodies.

### GC-MS analysis

Samples were analyzed using a model 7890A Agilent GC (Agilent Technologies, CA) equipped with a model 5975C MSD detector (Agilent Technologies), an autosampler (7683 B series, Agilent Technologies), a split/splitless injector, an injection module, and Chemstation software. The GC inlet temperature was set to 250°C with an injection volume of 1.0 µL and a split ratio of 1∶10, using helium as a carrier gas in constant-flow mode of 1.0 mL/min. A fused silica capillary column of 5% phenyl methylpolysiloxane phase (DB-5, Agilent Technologies) with dimensions 30 m×0.25 mm i.d. ×0.25 µm film thickness was used for analysis. The detector voltage was set to 1518 V, the auxiliary temperature was set to 280°C, the MS source temperature was set to 230°C, and the MS quad temperature was set to 150°C. The mass range was 50–700 Da. Data were obtained in full scan mode. The oven temperature for polar metabolite analysis was 80°C (hold 3 min) programmed to 130°C (3°C/min) then to 240°C (5°C/min) then to 320°C (10°C/min; hold 3 min). For the non-polar metabolite analysis, the detector voltage was set to 1588 V, and the mass range was 50–600 Da. The oven temperature was 80°C programmed to 260°C (5°C/min) then to 300°C (3°C/min; hold 3 min).

### Data analysis and enrichment analysis

Raw GC-MS data were processed as described by Styczynski *et al*. [31]to quantitatively compare global metabolites among all samples. Initially, the AMDIS (Automated Mass Spectral Deconvolution and Identification System, http://chemdata.nist.gov/mass-spc/amdis/) was used for mass spectral deconvolution, which separated peaks from noise and overlapping peaks. Then, the ELU files were subsequently analyzed with an online peak-filtering algorithm (SpectConnect, http://spectconnect.mit.edu). The identification was performed using spectra of individual components transferred to the NIST mass spectral search programs MS Search 2.0, where they were matched against the NIST MS library; for identification, a match quality of 70% was generally accepted. Peak areas of multiple derivative peaks belonging to one compound were summed and considered as a single compound. Normalization to an internal standard peak area was performed before multivariate statistical analyses.

The relative intensities of assigned metabolites by GC-MS analysis were analyzed in each sample. Significant differences in metabolite levels were detected by one-way analysis of variance (ANOVA) using PASW Statistics 18 software (IBM, Somers, NY) followed by Tukey's significant-difference test. The level of statistical significance was set at *p*<0.05. Partial least-squares discriminant analysis (PLS-DA) was performed in SIMCA-P software (version 12.0, Umetrics, Umeå, Sweden) using mean-centered and unit variance-scaled data, which yields a clearer differentiation of each class and enables a less complicated investigation of marker compounds than principal component analysis by rendering the class to each sample group [32]. Measures of model quality were reported for PLS-DA; the cumulative values of total Y explained variance (*R* ^2^), which describes the goodness of fit, and Y predictable variation (*Q* ^2^), which measures the predictive power of the model.

A data functional enrichment analysis with identified metabolic data for biological information was performed using MBRole [33], freely available from http://csbg.cnb.csic.es/mbrole/. The interface used ID conversion utility, which performs the analysis using the metabolites identified by KEGG (Kyoto Encyclopedia of Genes and Genomes; http://www.genome.jp/kegg/). We selected *Aspergillus niger* as a background set. The result contains the list annotation over-represented in the input set with respect to the background set and metabolite-associated p*-*values.

### Free radical scavenging activity

The freeze-dried *C. bassiana* (20 g) grown to different stages was extracted in screwcap vials with 400 mL of 70% methanol. The samples were irradiated four times in a microwave irradiation machine (MARSX, CEM Corporation, NC) for 10 min at 80°C. After extraction, the samples were filtered with filter paper (Whatman No. 4, Whatman, Kent, UK), freeze-dried (FDU-1200, EYELA, Miyagi, Japan) for 48 hours and then stored at −80°C for antioxidant activity analysis. The free radical scavenging ability of *C. bassiana* was determined by following the procedures by Kovatcheva-Apostolova et al. [34] with some modifications. The microwave extract sample solutions (10,000 mg/L) of 0.2 mL were mixed with 3.8 mL of 6×10^−5^ µM 2,2-diphenyl-1-picryl hydrazyl (DPPH) (Sigma, St. Louis, MO) solution. The mixture was incubated for 30 min in the dark at room temperature. The antioxidant activity of the plant was measured at 515 nm with a microplate spectrophotometer (xMark, Biorad, Berkeley, CA). Free radical scavenging activity (%) was calculated by the following formula:
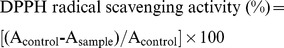



A_control_ is the absorbance without sample, and A_sample_ is the absorbance with sample.

## Results and Discussion

### Identification of metabolites in GC-MS data

Samples of fruit bodies at four developmental stages were extracted using 70% methanol and 100% *n*-hexane, respectively. Mass spectral data (Fig. S1) were identified from the NIST library. As listed in Table 1 and S1, the following 51 metabolites were identified in the 70% methanol extract of *C. bassiana* fruit bodies: alcohols (glucitol, inositol, mannitol), amino acids [alanine, γ-aminobutyric acid (GABA), aminoisobutyric acid, asparagine, aspartic acid, glutamine, glycine, histidine, homoserine, isoleucine, lysine, ornithine, proline, serine, threonine, tryptophan, tyrosine, and valine],organic acids (aconitic acid, citric acid, fumaric acid,gluconic acid, propanoic acid,isocitric acid, malic acid, mannonic acid, methylmalonic acid, and succinic acid), phosphoric acids (adenosine-5-monophosphoric acid and glycerophosphoric acid), purines (adenosine, guanosine, inosine, uracil, and uric acid), sugars (arabinose, fructose, galactose, glucose, glucuronic acid, maltose, mannose, *n*-acetylglucosamine, and ribose), and others (gulonolactone, nicotinic acid, and putrescine).

**Table 1:** Metabolites identified in 70% methanol extractsof *C. bassiana* fruiting bodies using GC-MS.

Class	Metabolite	RT	KEGG ID
**Alcohols**	Glucitol	32.57, 35.17, 36.57, 38.93, 41.25	C00794
	Inositol	34.38	C00137
	Mannitol	31.40, 35.26	C00392
**Amino acids**	Alanine	18.66	C01401
	γ-Aminobutyric acid	21.87	C00334
	Aminoisobutyric acid	18.06	C05145
	Asparagine	23.76, 25.63	C00152
	Aspartic acid	18.57, 21.69	C00049
	Glutamine	24.45	C00064
	Glycine	12.52, 14.00, 30.54	C00037
	Histidine	31.11	C00135
	Homoserine	19.48	C00263
	Isoleucine	13.56	C00407
	Lysine	31.21	C00047
	Ornithine	24.30, 27.45, 29.00	C00077
	Proline	13.67, 21.55	C00148
	Serine	16.23	C00065
	Threonine	17.20	C00188
	Tryptophan	36.64	C00078
	Tyrosine	31.60	C00082
	Valine	10.69	C00183
**Organic acids**	Aconitic acid	27.60	C00417
	Citric acid	29.15	C00158
	Fumaric acid	15.92	C00122
	Gluconic acid	32.67	C00257
	Propanoic acid	15.04	C00163
	Isocitric acid	29.16	C00311
	Malic acid	20.77	C00149
	Methylmalonic acid	19.04	C02170
	Succinic acid	14.46	C00042
**Phosphoric acids**	Adenosine 5'-monophosphate	45.99	C00020
	Glycerophosphoric acid	27.81, 33.80	C00093
**Purines**	Adenosine	42.48	C00212
	Guanosine	43.74	C00387
	Inosine	41.83	C00294
	Uracil	15.25	C00106
	Uric acid	34.65	C00366
**Sugars**	Arabinose	27.96, 30.76	C00259
	Fructose	30.12	C00095
	Galactose	30.53, 34.40, 38.24, 43.28	C00124
	Glucose	28.77, 32.37, 34.58, 38.43, 42.91	C00031
	Maltose	43.29, 44.06	C00208
	Mannose	28.78, 30.58, 39.96, 41.52	C00159
	N-acetylglucosamine*	34.88	C03878
	Ribose	38.24	C00121
**Others**	Gluconolactone	31.84	C00198
	Nicotinic acid	23.13	C00253
	Putrescine	27.13	C00134

* No annotation in enrichment analysis.

As listed in Table 2 and S2, 14 metabolites including saturated fatty acids (butyric acid, arachidic acid, behenic acid, lauric acid, lignoceric acid, margaric acid, myristic acid, and stearic acid), unsaturated fatty acid (linoleic acid, oleic acid, palmitoleic acid), and a fatty amide (oleamide), which had not been reported for *C. bassiana* fruiting bodies in previous studies by NMR [20],were identified in the 100% *n*-hexane extracts.

**Table 2:** Metabolites identified in 100% *n*-hexane extracts of *C. bassiana* fruiting bodies using GC-MS.

Class	Metabolite	RT	KEGG ID
**Saturated fatty acids**	Butyric acid	13.93	C00246
	Arachidic acid	34.24	C06425
	Behenic acid	37.34	C08281
	Lauric acid	19.33	C02679
	Lignoceric acid	40.60	C08320
	Margaric acid	29.22	NO ID
	Myristic acid	33.48, 23.54	C06424
	Palmitic acid	27.41	C00249
	Valeric acid*	25.51	C16537
	Stearic acid	30.96, 39.62	C01530
**Unsaturated fatty acids**	Linoleic acid	30.40, 32.01	C01595
	Oleic acid	30.49, 30.53, 30.62	C00712
	Palmitoleic acid	27.00	C08362
**Fatty amides**	Oleamide	33.76	NO ID

* No annotation in enrichment analysis.

### PLS-DA

PLS-DA was performed to compare the metabolic profiles of fruiting bodies at different developmental stages. The PLS-DA model was cross-validated. The goodness of fit and predictive ability of the PLS-DA model were quantified using *R* ^2^ *Y*, while the predictive ability of the model was indicated by *Q* ^2^ *Y*,respectively [35].The model performance parameters listed in Table 3;the results strongly suggest that the original models were valid. Both PLS-DA models had appropriate *R* ^2^ *Y* intercept values of 0.242 and 0.164, and *Q* ^2^ *Y* intercept values of −0.394, and −0.444, respectively. In general, models with an *R^2^Y* intercept of less than 0.4 and a *Q* ^2^ *Y* intercept of less than 0.05 are considered to be valid [32].

**Table 3:** Performance parameters of PLS-DA models and permutation tests.

PLS-DA model	R^2^Y	Q^2^Y	R^2^Y intercept	Q^2^Y intercept
70% methanol extract	0.967	0.895	0.242	−0.394
100% *n*-hexane extract	0.959	0.919	0.164	−0.444

R^2^Y is cumulative modeled variation in Y matrix.

Q^2^Y is cumulative predicted variation in the Y matrix.

The R^2^Y and Q^2^Y intercepts obtained after a permutation test (n = 999).

**Figure 1:**
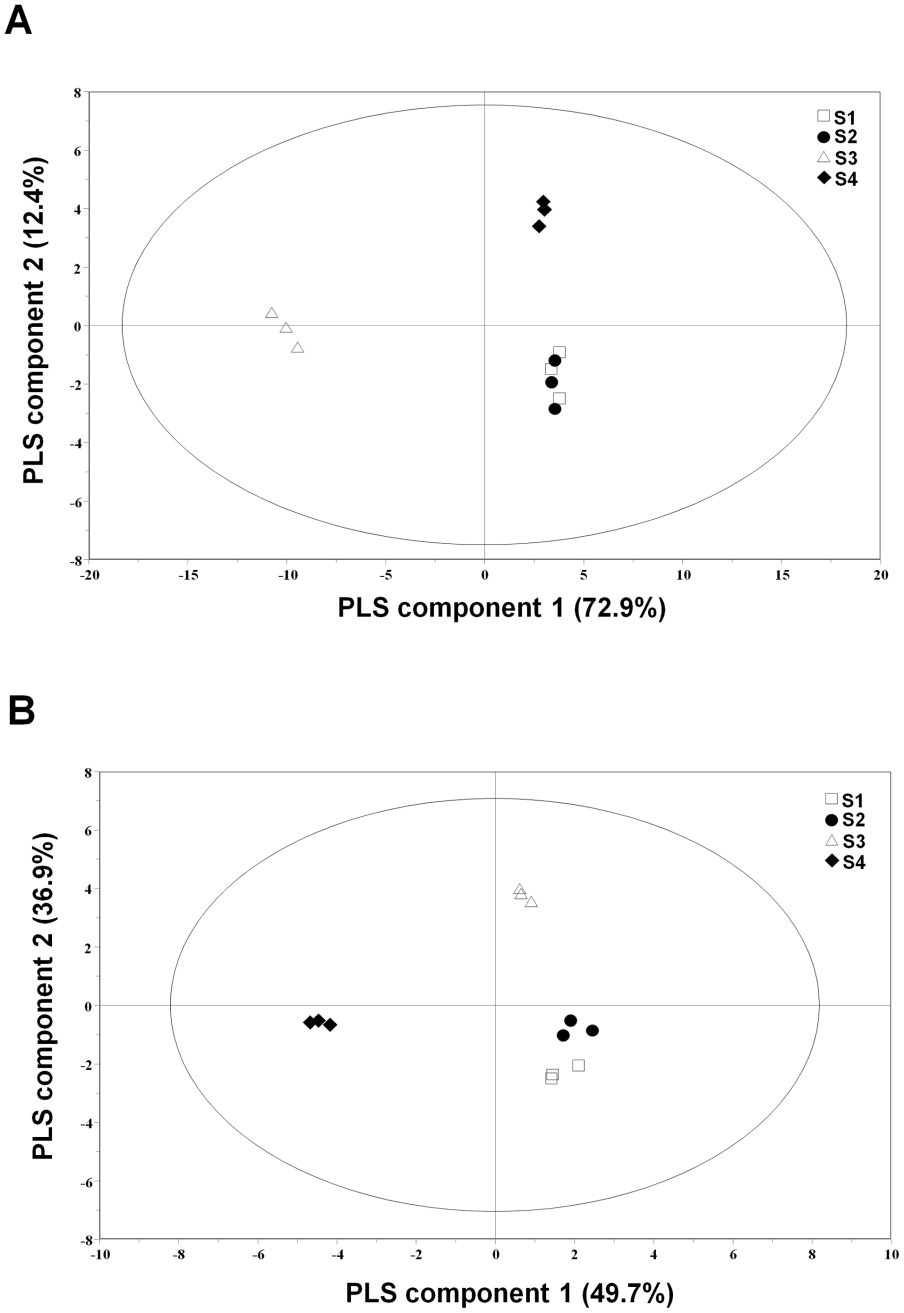
PLS-DA-derived score plots from 70% methanol (A), and 100% *n*-hexane (B) extracts of cultivated *C*. *bassiana* at various development stages. stage 1; □, stage 2; •, stage 3; △, stage 4; ♦.

The metabolic profile of the 70% methanol extracts of fruiting bodies at each developmental stage is represented in the PLS-DA-derived score plot as a single point (*R* ^2^ = 0.96, *Q* ^2^ = 0.89) in Figure 1A. Stage 3 samples were clearly separated from those samples of stages 1, 2, and 4 by principal component 1, which explained 72.9% of the variance. The samples of stages 1, 2, and 4 were separated mainly by principal component 2. Metabolic profiling of the PLS-DA score plot yielded similar findings to those obtained using the NMR data, but with different identified metabolites [20].As shown in Figure 1B, the samples from each condition could also be well separated in PLS-DA-derived score plots for 100% *n*-hexane extracts (*R* ^2^ = 0.95, *Q* ^2^ = 0.91). Samples from stage 4 were clearly separated from the other sample groups, mainly by PLS component 1, which indicates that profiles of non-polar metabolites in stage 4 samples were clearly distinguished from the samples at other stages.

Metabolic pathway analysis using enrichment analysis and ANOVA.

The most relevant metabolic pathways involved in the metabolite network during the development of *C. bassiana* fruit bodies were examined by annotating the identified metabolites by MBRole enrichment analysis (Table 4).Many metabolites overlapped in various metabolite annotations (Table S3). As listed in Table 4, the following 20 metabolites were associated with ATP-binding cassette (ABC) transporters (*p* = 0.0000): threonine, putrescine, serine, valine, fructose, arabinose, ribose, glucitol, isoleucine, lysine, glycine, ornithine, maltose, histidine, glutamine, glucose, aspartic acid, mannitol, glycerophosphoric acid, and proline. The following 13 were associated with ATP-binding cassette (ABC) transporters (*p* = 0.0000): tryptophan, proline, asparagine, tyrosine, aspartic acid, valine, isoleucine, serine, threonine, lysine, glutamine, histidine, and glycine. The role of aminoacyl-tRNA is to deliver amino acids to the ribosome for the biosynthesis of various proteins. Metabolites associated with purine metabolism (inosine, adenosine, AMP, uric acid, adenosine, guanosine, and glycine; *p = *0.0126), the TCA cycle (citric acid, fumaric acid, succinic acid, isocitric acid, aconitic acid, and malic acid; *p* = 0.0000) and biosynthesis and unsaturated fatty acids and fatty acid (lignoceric acid, lauric acid, myristic acid, stearic acid, arachidic acid, behenic acid, linoleic acid, palmitoleic acid, palmitic acid, and oleic acid, *p* = 0.0011 and 0.0030, respectively) were also described. This suggests that various metabolic pathways are associated with the different developmental stages of cultivated *C. bassiana*.

**Table 4:** List of KEGG pathways fromenrichment analysis of metabolite roles.

Interaction metabolite	*p*-value	Adjusted *p*-value	Pathway name	Hits
Threonine, putrescine, serine, valine, fructose, arabinose, ribose, glucitol, isoleucine, lysine, glycine, ornithine, maltose, histidine, glutamine, glucose, aspartic acid, mannitol, glycerophosphoric acid, proline	0.0000	0.0000	ABC transporters	20
Tryptophan, proline, asparagine, tyrosine, aspartic acid, valine, isoleucine, serine, threonine, lysine, glutamine, histidine, glycine	0.0000	0.0000	Aminoacyl-tRNA biosynthesis	13
Lignoceric acid, stearic acid, oleic acid, palmitic acid, arachidic acid, behenic acid, linoleic acid	0.0001	0.0011	Biosynthesis of unsaturated fatty acids	7
Aspartic acid, putrescine, gamma-aminobutyric acid, fumaric acid, glutamine, ornithine, proline	0.0016	0.0082	Arginine and proline metabolism	7
inosine, adenosine 5′-monophosphate, glutamine, uric acid, adenosine, guanosine, glycine	0.0031	0.0126	Purine metabolism	7
Citric acid, fumaric acid, succinic acid, isocitric acid, aconitic acid, malic acid	0.0000	0.0000	Citrate cycle (TCA cycle)	6
fumaric acid, gamma-aminobutyric acid, glutamine, succinic acid, aspartic acid, asparagine	0.0000	0.0001	Alanine, aspartate and glutamate metabolism	6
Fructose, glucose, myo-inositol, glucitol, galactose, mannose	0.0002	0.0014	Galactose metabolism	6
asparagine, aspartic acid, glycine, serine, tyrosine, alanine	0.0002	0.0014	Cyanoamino acid metabolism	6
Lauric acid, myristic acid, stearic acid, palmitoleic acid, palmitic acid, oleic acid	0.0005	0.0030	Fatty acid biosynthesis	6
Threonine, serine, glycine, tryptophan, aspartic acid, homoserine	0.0005	0.0030	Glycine, serine and threonine metabolism	6

*p*-value: statistically assessed against the background set.

Adjusted *p*-value: *p*-value corrected using the false discovery rate.

**Figure 2:**
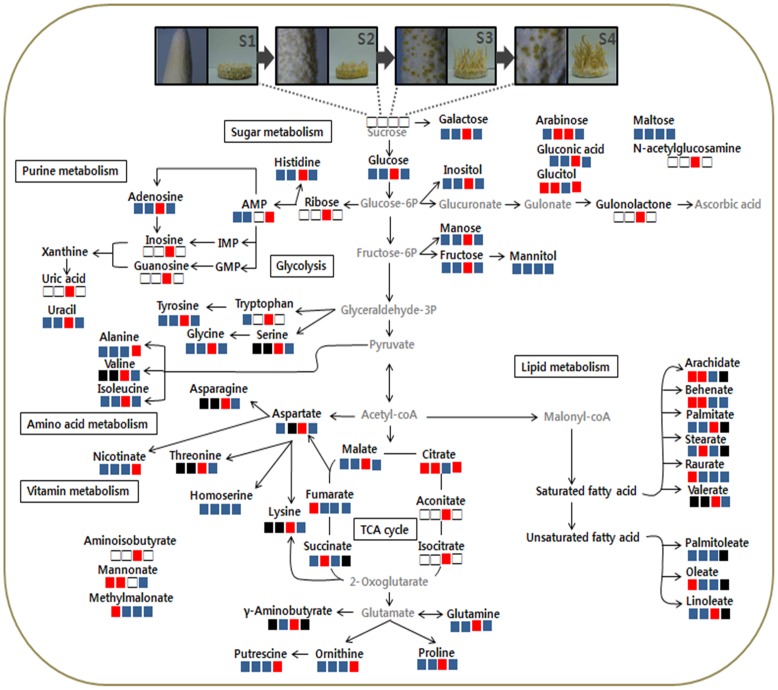
Relative levels of assigned metabolites in metabolic pathways during fruiting body development of cultivated *C*. *bassiana*. The relative levels of metabolites are indicated by various colors. significantly higher levels: red, lower levels: black, no significant differences: blue, not detected: white.

The results of the ANOVA conducted to compare differences in the relative levels of the metabolites at various developmental stages are presented in Tables S1 and S2. As shown in Fig. 2, significant changes in metabolites associated with sugar metabolism, purine metabolism, and amino acid metabolism were observed at stage 3. Most of the metabolites, threonine, serine, valine, fructose, arabinose, ribose, isoleucine, lysine, glycine, histidine, glutamine, glucose, and aspartic acid, were associated with ABC transporters and were present at a higher level in the completed perithecium formation stage (stage 3) than in the other stages. ABC-transporter proteins such as PDR, MDR, and MRP have been found in *Saccharomyces cerevisiae* and *Schizosaccharomyces pombe* [36]. It has been reported that the antioxidant capability in *B. bassiana* is reduced when ABC-transporter genes such as *Pdr*, *Mdr*, and *Mrp* are disrupted by mutation [37]. It is therefore expected that antioxidant activity will be higher at stage 3, in which higher levels of metabolites associated with ABC transporters were observed.

Energy-consuming ABC transporters exist in microbial cell membranes. These transporters play important roles in regulating nutrient uptake and secreting toxins or antimicrobial agents [38]–[40].It seems that various metabolites related to ABC transporters are needed and highly accumulate during stage 3 in order to regulate nutrient uptake or toxin secretion for ascospore formation. Besides ABC-transporters genes, the many transporters have been studied in various fungal species [41]–[43] However, to our knowledge the present report is the first regarding annotation of the metabolites related to ABC transporters in *C. bassiana* fruiting bodies at different development stages. Further studies on the roles of various transporters at different developmental stages of *C. bassiana* are needed.

Higher levels of amino acids associated with purine metabolism, such as glutamine and glycine, were observed at stage 3. Not only are the levels of amino acids such as tyrosine, tryptophan, asparagine and GABA increased at stage 3, but so also are those of purines such as adenosine, inosine, guanosine, and uric acid. Therefore, amino acid metabolism and purine metabolism may play important roles in regulating the development of cultivated *C.bassiana* fruiting bodies.

The levels of sugars such as fructose, galactose, glucose, and mannose were significantly increased at stage 3 (Fig. 2). A carbon source, such as glucose, is reported to act as an intermediate of glycolysis, the TCA cycle, and in the metabolism of purine, alanine, aspartate, and glutamate [44].Higher levels of the TCA-cycle intermediates aconitate and malate were also observed at stage 3, whereas the levels of citrate, succinate, and fumarate were lower.

As indicated in Figure 2 and Table S2, levels of saturated fatty acids such as arachidic acid, behenic acid, and lignoceric acid were higher levels in the no-perithecium (stage 1) and early perithecium formation (stage 2) stages, whereas both saturated and unsaturated fatty acids were decreased in the aging stage (stage 4). Up-regulated lipid biosynthesis gene expression has been reported during the early stages of perithecium development, together with a reduction in the levels and types of fatty acids [45]. Various lipids including linoleic acid were found to be associated with perithecium development in *Nectria haematococca* [46]. The lower levels of fatty acids observed in the present study in the cultivated *C. bassiana* at stage 4 may be attributable to the enhanced usage of the fatty acids as energy resources for ascospores release from the perithecia.

Among the various fatty acids, the levels of palmitic and linoleic acids which are known key intermediates involved in membrane biogenesis were higher level at stage 3 than at the other stages. It appears that palmitic and linoleic acids accumulate during stage 3 in preparation for ascospore formation.

Higher levels of putrescine and nicotinic acid, (also known as vitamin B_3_), were observed at stage 4. Putrescine is a breakdown product of amino acids [47] and is involved in the reduction in amino acids observed in stage 4 samples. It is interesting to note that the levels of most of the fatty acids had decreased at stage 4, whereas that of nicotinic acid increased. It may be that the fatty-acid breakdown products are converted into putrescine and nicotinic acid during fruiting body formation.

To summarize, it appears that various metabolites are needed for the formation of ascospore in the perithecium in stage 3. Transcriptomic and proteomic approaches will be applied in the near future to investigate or confirm the mechanism underlying the variations in metabolic profiles that occur during the various development stages of the *C. bassiana* fruiting body.

### DPPH radical-scavenging activity

**Figure 3:**
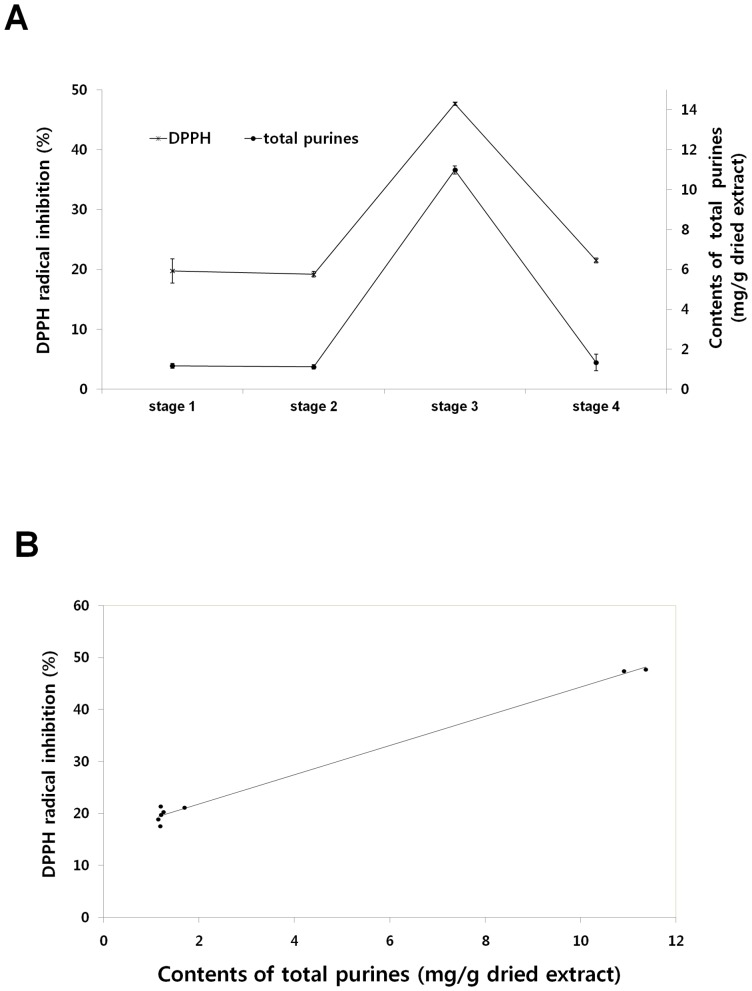
Free radical scavenging activities and total purine contents (A) and correlation (B) to various development stages of cultivated *C*. *bassiana*.

Variations in the DPPH radical-scavenging activities during the development of *C. bassiana* fruiting bodies were investigated; the results are summarized in Table 5. The free-radical-scavenging activities of stages 1, 2, and 4 were similar, whereas those of stage 3 were significantly higher. Ascorbic acid was used as a positive control. For antioxidant activity, the DPPH radical-scavenging activity of stage 3 (10,000 mg/L) was 47.7%, while that of ascorbic acid (50 mg/L) was 41.1%.In *C. bassiana*, the total phenol content was higher in stage 3 samples (i.e., after 6 weeks of cultivation) than in samples from other stages [48]. It has been reported that the activities of superoxide dismutase and catalase in *B. bassiana* prevent an increase in reactive oxygen species and the subsequent damage that they can cause [14].Purine ribonucleosides, including guanosine, inosine, and adenosine, have been reported to protect against DNA oxidative damage by reducing the production of hydrogen peroxide and hydroxyl radicals [49].Furthermore, uric acid, which scavenges hydroxyl radicals, is known to be an important antioxidant [50]. Adenosine, guanosine, inosine, and uric acid, the levels of which are enhanced at stage 3, are associated with antioxidant activity. As shown in Figure 3, strong positive correlations (coefficients *r* ^2^ = 0.99) were found in the present study between the content of purine nucleoside compounds (Table S4) and antioxidant activities (Table 5). Adenosine is also known to act as an antiwrinkle compound for human skin [51], [52].Thus, cultivated fruit bodies of *C. bassiana* at stage 3 might be considered natural resources for nutraceuticals with free-radical scavenging activity or as cosmeceutical materials with antiwrinkle activity.

**Table 5:** The free-radical scavenging activity according to developmental stage of *C. bassiana* (10,000 mg/L).

Sample	DPPH radical inhibition (%)
Stage 1	19.7±1.6^a^
Stage 2	19.2±0.4^a^
Stage 3	47.7±0.2^b^
Stage 4	21.5±0.3^a^
Ascorbic acid (50 mg/L)	41.1±0.8^c^

Different letters in the same column indicate significant differences at *p*<0.05. Data are mean ± STD values for triplicate measurements.

## Conclusions

In this study, we performed metabolic profiling of *C. bassiana* using a GC-MS-based non-targeted profiling approach. Alteration of several major metabolic pathways, including sugar metabolism, purine metabolisms, amino acid metabolism, TCA cycle, and lipid metabolism, were observed during fruiting body development. Especially, in the perithecium formation stage (stage 3), the relative levels of metabolites associated with ABC transporters, aminoacyl-tRNA biosynthesis, and purine metabolism were significantly increased, whereas levels of metabolites associated with lipid metabolism decreased in the ascospores released stage (stage 4).The free radical scavenging activity, which was significantly higher in stage 3 than in the other stages, was positively correlated with purine derivatives such as adenosine, guanosine, inosine and uric acid. Thus, *C. bassiana* of stage 3 was suggested to be a better resource with higher level of free-radical scavenging activities compared to the other fruiting body samples of *C. bassiana* at other stages. We suggest that the metabolic profiles during fruiting body development determined in this study provide useful criteria for selecting appropriate harvest points of fruiting bodies of *C. bassiana*. To our knowledge, this is the first study to investigate the correlation between metabolic profiles and free radical scavenging activities of *C. bassiana* fruiting bodies according to developmental stage.

## Supporting Information

Figure S1 **Representative GC-MS spectra of 70% methanol (A), and 100%** ***n*** **-hexane (B) extracts of cultivated** ***C*** **.** ***bassiana*** **at various development stages.**
(TIF)

Table S1 **Metabolites identified by GC-MS analysis of 70% methanol extracts in** ***C*** **.** ***bassiana*** **fruiting bodies.** The relative levels of each metabolite were obtained by dividing the area % of metabolite by the area % of internal standard. Different letters in the same row represent a significant difference. Data are mean ± STD values for triplicate measurements. ND, not detected in the sample.
(DOCX)

Table S2 **Metabolite identified by GC-MS analysis of 100% n-hexane extracts of** ***C*** **.** ***bassiana*** **fruiting bodies.** The relative levels of each metabolite were obtained by dividing the area % of metabolite by the area % of internal standard. Different letters in the same row represent a significant difference. Data are mean ± STD values for triplicate measurements. ND, not detected in the sample.
(DOCX)

Table S3 **Additional list of KEGG pathways from enrichment analysis of metabolite roles.**
(DOCX)

Table S4 **Quantification of purine contents according to developmental stage of** ***C*** **.** ***bassiana*** **.** Data are mean ± STD values for duplicate measurements. Different letters in the same row represent a significant difference.
(DOCX)
