# Supplementary material for: Metabolic Profiles and Free Radical Scavenging Activity of Cordyceps bassiana Fruiting Bodies According to Developmental Stage
Source: PLoS One. 2013 Sep 13;8(9):e73065. doi: 10.1371/journal.pone.0073065 (PMC3772819; doi:10.1371/journal.pone.0073065)
Supplement: Table S1 — Metabolites identified by GC-MS analysis of 70% methanol extracts in C . bassiana fruiting bodies. The relative levels of each metabolite were obtained by dividing the area % of metabolite by the area % of internal standard. Different letters in the same row represent a significant difference. Data are mean ± STD values for triplicate measurements. ND, not detected in the sample. (DOCX) [file pone.0073065.s002.docx]

**Table S1. Metabolites identified by GC-MS analysis of 70% methanol extracts in *C. bassiana* fruiting bodies.** The relative levels of each metabolite were obtained by dividing the area % of metabolite by the area % of internal standard. Different letters in the same row represent a significant difference. Data are mean±STD values for triplicate measurements. ND, not detected in the sample.

| Compound | RT | Relative level of samples | | | |
| --- | --- | --- | --- | --- | --- |
|  |  | Stage1 | Stage2 | Stage3 | Stage4 |
| **Alcohols** |  |  |  |  |  |
| Glucitol | 32.57 | 0.91±0.12^a^ | 0.91±0.07^a^ | 0.65±0.02^b^ | 0.98±0.04^a^ |
|  | 35.17 |  |  |  |  |
|  | 36.57 |  |  |  |  |
|  | 38.93 |  |  |  |  |
|  | 41.25 |  |  |  |  |
| Inositol | 34.38 | 1.62±0.03^a^ | 1.67±0.13^a^ | 2.13±0.15^b^ | 2.11±0.16^b^ |
| Mannitol | 31.40  35.26 | 52.30±21.16^a^ | 88.43±44.19^a^ | 87.07±17.76^a^ | 83.27±17.43^a^ |
| **Amino acids** |  |  |  |  |  |
| Alanine | 18.66 | 0.08±0.01^a^ | 0.07±0.03^a^ | 0.10±0.01^ab^ | 0.13±0.01^b^ |
| γ- Aminobutyric acid | 21.87 | 0.20±0.02^a^ | 0.27±0.03^b^ | 0.56±0.02^c^ | 0.19±0.01^a^ |
| Aminoisobutyric acid | 18.06 | ND | ND | 0.11±0.01 | ND |
| Asparagine | 23.76  25.63 | 0.12±0.03^ac^ | 0.09±0.001^a^ | 1.24±0.02^b^ | 0.16±0.01^c^ |
| Aspartic acid | 18.57  21.69 | 0.77±0.15^a^ | 0.42±0.03^b^ | 3.01±0.16^c^ | 0.72±0.04^a^ |
| Glutamine | 24.45 | 11.08±2.44^a^ | 8.54±0.59^a^ | 22.23±3.82^b^ | 13.58±0.61^a^ |
| Glycine | 12.52  14.00  30.54 | 2.50±0.04^a^ | 2.43±0.35^a^ | 11.18±0.32^b^ | 3.06±0.17^a^ |
| Histidine | 31.11 | 0.23±0.02^a^ | 0.15±0.04^a^ | 1.29±0.16^b^ | 0.23±0.03^a^ |
| Homoserine | 19.48 | 0.05±0.01^a^ | 0.06±0.001^ab^ | 0.07±0.01^b^ | 0.06±0.001^ab^ |
| Isoleucine | 13.56 | 0.40±0.15^a^ | 0.43±0.02^a^ | 5.74±1.90^b^ | 0.59±0.03^a^ |
| Lysine | 31.21 | 0.75±0.38^ac^ | 0.65±0.07^a^ | 3.44±0.15^b^ | 1.29±0.07^c^ |
| Ornithine | 24.30  27.45  29.00 | 10.53±1.76^a^ | 10.58±0.85^a^ | 9.59±0.43^a^ | 13.49±0.90^b^ |
| Proline | 13.67  21.55 | 12.73±5.56^a^ | 13.41±0.84^a^ | 25.26±0.51^b^ | 17.10±0.57^a^ |
| Serine | 16.23 | 1.65±0.45^a^ | 1.56±0.10^a^ | 5.90±0.18^b^ | 2.47±0.12^c^ |
| Threonine | 17.20 | 1.29±0.23^a^ | 1.14±0.05^a^ | 3.26±0.05^b^ | 2.32±0.08^c^ |
| Tryptophan | 36.64 | 0.05±0.00^a^ | ND | 0.14±0.01^b^ | ND |
| Tyrosine | 31.60 | 0.82±0.38^a^ | 0.83±0.06^a^ | 5.66±0.34^b^ | 1.09±0.09^a^ |
| Valine | 10.69 | 0.98±0.32^a^ | 0.94±0.04^a^ | 6.91±0.22^b^ | 1.52±0.08^c^ |
| **Organic acids** |  |  |  |  |  |
| Aconitic acid | 27.60 | ND | ND | 0.07±0.01 | ND |
| Citric acid | 29.15 | 18.25±0.98^ab^ | 20.31±1.01^b^ | 12.74±0.77^c^ | 17.93±0.82^a^ |
| Fumaric acid | 15.92 | 1.13±0.06^a^ | 0.70±0.06^b^ | 0.78±0.03^b^ | 0.85±0.08^b^ |
| Gluconic acid | 32.67 | 0.36±0.10^a^ | 0.30±0.01^a^ | 4.94±0.17^b^ | 0.40±0.02^a^ |
| Propanoic acid | 15.04 | 0.03±0.00^a^ | 0.03±0.00^a^ | 0.50±0.02^b^ | 0.03±0.00^a^ |
| Isocitric acid | 29.16 | ND | ND | 0.31±0.02 | ND |
| Malic acid | 20.77 | 3.35±0.10^a^ | 3.82±0.32^a^ | 7.84±0.29^b^ | 3.31±0.13^a^ |
| Methylmalonic acid | 19.04 | 0.16±0.00^a^ | 0.12±0.03^b^ | 0.13±0.01^ab^ | 0.11±0.01^b^ |
| Succinic acid | 14.46 | 2.24±0.09^a^ | 3.41±0.16^b^ | 2.15±0.06^a^ | 1.80±0.08^c^ |
| **Phosphoric acids** |  |  |  |  |  |
| Adenosine 5'-monophosphate | 45.99 | 0.35±0.05^ab^ | 0.30±0.04^a^ | ND | 0.44±0.08^b^ |
| Glycerophosphoric acid | 27.81  33.80 | 1.53±0.36^ab^ | 1.79±0.12^ab^ | 1.41±0.09^a^ | 2.05±0.14^b^ |
| **Purines** |  |  |  |  |  |
| Adenosine | 42.48 | 0.05±0.005^a^ | 0.04±0.004^a^ | 2.66±0.11^b^ | 0.04±0.00^a^ |
| Guanosine | 43.74 | ND | ND | 1.06±0.07 | ND |
| Inosine | 41.83 | ND | ND | 0.12±0.01 | ND |
| Uracil | 15.25 | 0.02±0.001^a^ | 0.03±0.01^a^ | 0.38±0.03^b^ | 0.02±0.00^a^ |
| Uric acid | 34.65 | ND | ND | 0.06±0.001 | ND |
| **Sugars** |  |  |  |  |  |
| Arabinose | 27.96  30.76 | 0.14±0.01^a^ | 0.10±0.02^b^ | 0.10±0.01^b^ | 0.16±0.01^a^ |
| Fructose | 30.12 | 0.06±0.02^a^ | 0.07±0.00^a^ | 0.78±0.02^b^ | 0.06±0.01^a^ |
| Galactose | 30.53  34.40  38.24  43.28 | 0.42±0.04^a^ | 0.55±0.03^a^ | 1.57±0.25^b^ | 0.40±0.04^a^ |
| Glucose | 28.77  32.37  34.58  38.43  42.91 | 1.25±1.11^a^ | 1.52±0.45^a^ | 32.44±1.84^b^ | 2.28±0.69^a^ |
| Maltose | 43.29  44.06 | 0.19±0.09^a^ | 0.38±0.22^a^ | 0.32±0.01^a^ | 0.46±0.17^a^ |
| Mannose | 28.78  30.58  39.96  41.52 | 0.81±0.03^a^ | 0.68±0.04^a^ | 21.80±0.91^b^ | 0.84±0.14^a^ |
| N-acetylglucosamine | 34.88 | ND | ND | 1.04±0.03 | ND |
| Ribose | 38.24 | ND | ND | 0.32±0.01 | ND |
| **Others** |  |  |  |  |  |
| Gluconolactone | 31.84 | 0.20±0.01^a^ | 0.22±0.02^a^ | 0.25±0.02 ^a^ | 0.16±0.01^b^ |
| Nicotinic acid | 23.13 | 0.18±0.00^a^ | 0.16±0.01^a^ | 0.17±0.01^a^ | 0.20±0.00^b^ |
| Putrescine | 27.13 | 3.94±0.73^a^ | 3.86±0.43^a^ | 4.79±0.11^a^ | 5.78±0.15^b^ |
