# Supplementary material for: Metabolic Profiles and Free Radical Scavenging Activity of Cordyceps bassiana Fruiting Bodies According to Developmental Stage
Source: PLoS One. 2013 Sep 13;8(9):e73065. doi: 10.1371/journal.pone.0073065 (PMC3772819; doi:10.1371/journal.pone.0073065)
Supplement: Table S2 — Metabolite identified by GC-MS analysis of 100% n-hexane extracts of C . bassiana fruiting bodies. The relative levels of each metabolite were obtained by dividing the area % of metabolite by the area % of internal standard. Different letters in the same row represent a significant difference. Data are mean ± STD values for triplicate measurements. ND, not detected in the sample. (DOCX) [file pone.0073065.s003.docx]

**Table S2. Metabolite identified by GC-MS analysis of 100%** *n***-hexane extracts of *C. bassiana* fruiting bodies.** The relative levels of each metabolite were obtained by dividing the area % of metabolite by the area % of internal standard. Different letters in the same row represent a significant difference. Data are mean±STD values for triplicate measurements. ND, not detected in the sample.

| Compound | RT | Relative level of samples | | | |
| --- | --- | --- | --- | --- | --- |
|  |  | Stage1 | Stage2 | Stage3 | Stage4 |
| **Saturated fatty acids** |  |  |  |  |  |
| Butyric acid | 13.93 | 0.90±0.04^a^ | 0.89±0.01^ab^ | 0.88±0.05^ab^ | 0.79±0.04^b^ |
| Arachidic acid | 34.24 | 0.89±0.02^a^ | 0.87±0.04^a^ | 0.65±0.01^b^ | 0.46±0.05^c^ |
| Behenic acid | 37.34 | 0.79±0.04^a^ | 0.78±0.04^a^ | 0.52±0.06^b^ | 0.38±0.10^b^ |
| Lauric acid | 19.33 | 0.65±0.02^a^ | 0.54±0.01^b^ | 0.50±0.02^b^ | 0.51±0.04^b^ |
| Lignoceric acid | 40.60 | 0.98±0.02^a^ | 1.23±0.07^b^ | 0.84±0.09^a^ | 0.58±0.06^c^ |
| Margaric acid | 29.22 | 0.44±0.04^a^ | 0.54±0.07^ab^ | 1.75±0.11^c^ | 0.63±0.04^b^ |
| Myristic acid | 33.48  23.54 | 2.66±0.01^a^ | 2.58±0.10^ab^ | 2.38±0.10^b^ | 2.13±0.10^c^ |
| Palmitic acid | 27.41 | 21.40±0.45^a^ | 25.94±0.79^b^ | 59.34±1.85^c^ | 17.05±0.55^d^ |
| Valeric acid | 25.51 | 0.25±0.04^a^ | 0.26±0.01^a^ | 1.59±0.06^b^ | 0.37±0.02^c^ |
| Stearic acid | 30.96  39.62 | 58.79±1.49^a^ | 64.31±1.81^b^ | 60.54±1.95^ab^ | 50.94±2.35^c^ |
| **Unsaturated fatty acids** |  |  |  |  |  |
| Linoleic acid | 30.40  32.01 | 32.11±0.61^a^ | 34.14±0.26^b^ | 46.45±0.97^c^ | 24.42±1.01^d^ |
| Oleic acid | 30.49  30.53  30.62 | 11.10±0.81^a^ | 8.42±0.81^b^ | 6.55±0.15^c^ | 3.78±0.42^d^ |
| Palmitoleic acid | 27.00 | 0.31±0.03^a^ | 0.32±0.03^a^ | 0.30±0.01^a^ | 0.18±0.01^b^ |
| **Fatty amides** |  |  |  |  |  |
| Oleamide | 33.76 | 0.35±0.01^a^ | 0.75±0.02^ab^ | 1.27±0.60^b^ | 0.38±0.01^a^ |
