# Supplementary material for: Metabolic Profiles and Free Radical Scavenging Activity of Cordyceps bassiana Fruiting Bodies According to Developmental Stage
Source: PLoS One. 2013 Sep 13;8(9):e73065. doi: 10.1371/journal.pone.0073065 (PMC3772819; doi:10.1371/journal.pone.0073065)
Supplement: Table S3 — Additional list of KEGG pathways from enrichment analysis of metabolite roles. (DOCX) [file pone.0073065.s004.docx]

**Table S3.** Additional list of KEGG pathways from enrichment analysis of metabolite roles.

| Interaction metabolite | *p-*value | Adjusted *p*-value | Pathway name | Hits |
| --- | --- | --- | --- | --- |
| Malic acid, isocitric acid, aconitic acid, succinic acid, citric acid | 0.0022 | 0.0096 | Glyoxylate and dicarboxylate metabolism | 5 |
| Glycine, asparagine, glutamine, aspartic acid | 0.0020 | 0.0095 | Nitrogen metabolism | 4 |
| Uracil, gamma-aminobutyric acid, histidine, aspartic acid | 0.0040 | 0.0148 | beta-Alanine metabolism | 4 |
| Glucose, gluconic acid, ribose, Glucon lactone | 0.0045 | 0.0156 | Pentose phosphate pathway | 4 |
| Succinic acid, propanoic acid, methylmalonic acid, valine | 0.0069 | 0.0226 | Propanoate metabolism | 4 |
| Butyric acid, fumaric acid, succinic acid, gamma-aminobutyric acid | 0.0100 | 0.0310 | Butanoate metabolism | 4 |
| Aspartic acid, fumaric acid, nicotinic acid, propanoic acid | 0.0139 | 0.0410 | Nicotinate and nicotinamide metabolism | 4 |
| Mannitol, glucitol, mannose, fructose | 0.0187 | 0.0514 | Fructose and mannose metabolism | 4 |
| Methylmalonic acid, aminoisobutyric acid, glutamine, uracil | 0.0367 | 0.0857 | Pyrimidine metabolism | 4 |
| Glucose, mannose, galactose, arabinose | 0.1159 | 0.1949 | Amino sugar and nucleotide sugar metabolism | 4 |
| Valine, uracil, aspartic acid | 0.0193 | 0.0514 | Pantothenate and CoA biosynthesis | 3 |
| Valine, threonine, isoleucien | 0.0213 | 0.0541 | Valine, leucine and isoleucine biosynthesis | 3 |
| Aspartic acid, lysine, homoserine | 0.0303 | 0.0738 | Lysine biosynthesis | 3 |
| Glycine, ornithine, putrescine | 0.0471 | 0.1014 | Glutathione metabolism | 3 |
| Valine, isoleucine, methylmalonic acid | 0.0569 | 0.1138 | Valine, leucine and isoleucine degradation | 3 |
| Tyrosine, fumaric acid, succinic acid | 0.0752 | 0.1452 | Phenylalanine metabolism | 3 |
| Glucose, fructose, maltose | 0.0914 | 0.1707 | Starch and sucrose metabolism | 3 |
| Aspartic acid, Serine, homoserine | 0.1183 | 0.1949 | Cysteine and methionine metabolism | 3 |
| Succinic acid, tyrosine, fumaric acid | 0.2242 | 0.3487 | Tyrosine metabolism | 3 |
| Succinic acid, fumaric acid | 0.0452 | 0.1012 | Oxidative phosphorylation | 2 |
| Homoserine, serine | 0.0561 | 0.1138 | Sulfur metabolism | 2 |
| Tyrosine, glycine | 0.1068 | 0.1930 | Thiamine metabolism | 2 |
| Tryptophan, tyrosine | 0.1138 | 0.1949 | Phenylalanine, tyrosine and tryptophan biosynthesis | 2 |
| Serine, glycine | 0.1657 | 0.2651 | Methane metabolism | 2 |
| Aspartic acid, histidine | 0.2449 | 0.3707 | Histidine metabolism | 2 |
| Glycine, lysine | 0.2692 | 0.3865 | Lysine degradation | 2 |
| Myo-inositol, arabinose | 0.2692 | 0.3865 | Ascorbate and aldarate metabolism | 2 |
| Glycine, threonine | 0.7650 | 1.0000 | Porphyrin and chlorophyll metabolism | 2 |

*p*-value: statistically assessed against the background set

Adjusted *p*-value: *p*-value corrected using the false discovery rate.
