# Supplementary material for: Metabolic Profiles and Free Radical Scavenging Activity of Cordyceps bassiana Fruiting Bodies According to Developmental Stage
Source: PLoS One. 2013 Sep 13;8(9):e73065. doi: 10.1371/journal.pone.0073065 (PMC3772819; doi:10.1371/journal.pone.0073065)
Supplement: Table S4 — Quantification of purine contents according to developmental stage of C . bassiana . Data are mean ± STD values for duplicate measurements. Different letters in the same row represent a significant difference. (DOCX) [file pone.0073065.s005.docx]

**Table S4. Quantification of purine contents according to developmental stage of *C. bassiana*.** Data are mean±STD values for duplicate measurements. Different letters in the same row represent a significant difference.

| Compound | Quant. ion^§^ (m/z) | *R*^2^ | Regression equation | Contents in sample (mg/g dried extract) | | | |
| --- | --- | --- | --- | --- | --- | --- | --- |
|  |  |  |  | Stage 1 | Stage 2 | Stage 3 | Stage 4 |
| Adenosine | 230 | 0.991 | Y=0.0209x-0.2212 | 1.17±0.12^a^ | 1.12±0.11^a^ | 4.50±0.39^b^ | 1.34±0.41^a^ |
| Guanosine | 324 | 0.995 | Y=0.0049x-0.0217 | nd | nd | 4.81±0.40 | nd |
| Inosine | 217 | 0.994 | Y=0.0097x-0.0251 | nd | nd | 1.07±0.01 | nd |
| Uric acid | 441 | 0.991 | Y=0.0231x-0.1347 | nd | nd | 0.60±0.01 | nd |

Quant. ion: characteristic ion for quantification
